# Complement C5a Receptor Signaling Alters Stress Responsiveness and Modulates Microglia Following Chronic Stress Exposure

**DOI:** 10.1016/j.bpsgos.2024.100306

**Published:** 2024-03-07

**Authors:** Hsiao-Jou Cortina Chen, Jereme G. Spiers, Titaya Lerskiatiphanich, Sandra E. Parker, Nickolas A. Lavidis, Jenny N. Fung, Trent M. Woodruff, John D. Lee

**Affiliations:** a School of Biomedical Sciences, the University of Queensland, St. Lucia, Brisbane, Queensland, Australia; b Metabolic Research Laboratories, Wellcome Trust MRC Institute of Metabolic Science, University of Cambridge, Addenbrooke’s Hospital, Cambridge, United Kingdom; c Department of Biochemistry and Genetics, La Trobe Institute for Molecular Science, La Trobe University, Bundoora, Victoria, Australia; d Clear Vision Research, Eccles Institute of Neuroscience, John Curtin School of Medical Research, College of Health and Medicine, the Australian National University, Acton, Australian Capital Territory, Australia; e School of Medicine and Psychology, College of Health and Medicine, the Australian National University, Australian Capital Territory, Australia; f Queensland Brain Institute, the University of Queensland, St Lucia, Brisbane, Queensland, Australia

**Keywords:** C5a receptor, Complement system, Corticosterone, Glia, Hippocampus, Neuroinflammation

## Abstract

**Background:**

Accumulating evidence underscores the pivotal role of heightened inflammation in the pathophysiology of stress-related diseases, but the underlying mechanisms remain elusive. The complement system, a key effector of the innate immune system, produces the C5–cleaved activation product C5a upon activation, initiating inflammatory responses through the canonical C5a receptor 1 (C5aR1). While C5aR1 is expressed in stress-responsive brain regions, its role in stress responsiveness remains unknown.

**Methods:**

To investigate C5a-C5aR1 signaling in stress responses, mice underwent acute and chronic stress paradigms. Circulating C5a levels and messenger RNA expression of C5aR1 in the hippocampus and adrenal gland were measured. C5aR1-deficient mice were used to elucidate the effects of disrupted C5a-C5aR1 signaling across behavioral, hormonal, metabolic, and inflammation parameters.

**Results:**

Chronic restraint stress elevated circulating C5a levels while reducing C5aR1 messenger RNA expression in the hippocampus and adrenal gland. Notably, the absence of C5aR1 signaling enhanced adrenal sensitivity to adrenocorticotropic hormone, concurrently reducing pituitary adrenocorticotropic hormone production and enhancing the response to acute stress. C5aR1-deficient mice exhibited attenuated reductions in locomotor activity and body weight under chronic stress. Additionally, these mice displayed increased glucocorticoid receptor sensitivity and disrupted glucose and insulin homeostasis. Chronic stress induced an increase in C5aR1-expressing microglia in the hippocampus, a response mitigated in C5aR1-deficient mice.

**Conclusions:**

C5a-C5aR1 signaling emerges as a key metabolic regulator during stress, suggesting that complement activation and dysfunctional C5aR1 signaling may contribute to neuroinflammatory phenotypes in stress-related disorders. The results advocate for further exploration of complement C5aR1 as a potential therapeutic target for stress-related conditions.

## Plain Language Summary

How the immune system, particularly the complement system, influences responses to stress has not been fully clear. In this study, we focus on C5a-C5aR1 signaling, a part of the immune system, and found that it significantly affects stress-related reactions in mice. In chronic stress, we observed increased inflammation, altered hormonal responses, and disrupted metabolic regulation. Mice lacking C5aR1 showed reduced stress-induced behavioral changes, indicating that this receptor may play a vital role in modulating the stress response. Understanding these immune mechanisms sheds light on stress-related disorders and may open avenues for therapeutic interventions.

Increasing evidence suggests that the etiopathogenesis of neuropsychiatric disorders is strongly associated with abnormal immunological mechanisms, with prolonged immune activation and resultant release of proinflammatory cytokines underpinning the behavioral and neuroendocrine alterations present in these conditions (1, 2, 3, 4, 5, 6). Some of the earliest observed effects of these proinflammatory cytokines include direct modulation of the hypothalamic-pituitary-adrenal (HPA) axis, the pathway regulating glucocorticoid synthesis (7,8). Cytokines including interleukin (IL) 1β and IL-6 stimulate the expression and release of hypothalamic corticotropin-releasing hormone and pituitary-derived adrenocorticotropic hormone (ACTH), which induce de novo production of adrenal glucocorticoids (9, 10, 11). However, the relationship between stress-induced glucocorticoids and immunity is complex, with glucocorticoids exhibiting either pro- or anti-inflammatory effects depending on a multitude of factors including tissue, cell type, and duration of exposure. Prolonged activation of the HPA axis can lead to disruption of glucocorticoid negative feedback and promote glucocorticoid resistance within the immune system, resulting in sustained inflammation that fundamentally underpins neuropsychiatric conditions such as major depression (12,13).

In addition to the immunomodulatory effects of proinflammatory cytokines on the response to stress, expression of several innate immune receptors has been observed in stress-related tissues (14). Notably, this includes members of the complement cascade, a chief component of the innate immune surveillance system mediating host homeostasis, inflammation, and defense against invading pathogens (15,16). The most potent inflammatory member of this pathway, complement component C5a, elicits several important biological effects including modulation of inflammatory cell chemotaxis, cellular respiratory burst, cytokine and chemokine release, and phagocytosis (17). These potent inflammatory functions of C5a are mediated by two 7-transmembrane receptors of the rhodopsin family, C5a receptor 1 (C5aR1) and C5aR2 (18). The majority of proinflammatory effects of C5a result from binding to the canonical G protein–coupled C5aR1 (19), and accumulating evidence has implicated an overproduction of C5a or overexpressed C5aR1 in the pathogenesis of many inflammatory conditions, autoimmune dysfunction, and neurodegenerative diseases (20, 21, 22, 23).

Previous work demonstrated that C5aR1 is localized to the anterior pituitary gland and plays a role in regulating inflammation (24). In addition to this inflammatory function, C5a-C5aR1 signaling was shown to independently stimulate ACTH release, thereby regulating the release of adrenal corticosteroids. In conditions of stress-related psychopathology, dysfunction of complement has been linked with posttraumatic stress disorder and schizophrenia susceptibility (25,26). However, the relationship between in vivo C5a-C5aR1 signaling and stress responsiveness has not been studied. Therefore, in the current study, we aimed to determine whether C5aR1 signaling can modulate the neuroendocrine response to stress and further investigated the behavioral and molecular mechanisms during the transition from acute to chronic stress using C5aR1-deficient mice. We showed that C5aR1 signaling is required for nominal ACTH release following acute stress exposure, with loss of this signaling reducing circulating ACTH concentrations and increasing adrenal sensitivity. Under conditions of chronic stress, loss of C5aR1 signaling alters stress-induced glucose production, primarily by increasing insulin secretion. In addition, chronic stress increases the number of immunoreactive microglia, an effect that is attenuated in the absence of C5aR1 signaling. This general reduction in stress-induced microglial reactivity highlights C5-C5aR1 as a novel nonsteroidal anti-inflammatory target that would be clinically advantageous in conditions for which stress and glucocorticoids are underlying contributors to inflammation.

## Methods and Materials

### Experimental Animals

Wild-type (WT) C57BL/6J male mice ages 10 to 11 weeks were obtained from the Animal Resources Centre in Canning Vale, Australia. Homozygous C5aR1-deficient mice (C5aR1^−/−^) were generated and bred as previously described (27). To generate experimental animals, WT and C5aR1^−/−^ mice were bred to generate heterozygous mice. These heterozygous mice were further crossed, and male homozygous C5aR1^−/−^ and WT littermate control mice were used in experimental studies. Mice were housed within the University of Queensland animal facility under a 12-hour light/dark cycle (on at 6:00 am), with room temperature maintained at 24 ± 2 °C. To verify the absence of C5aR1, genotyping was performed on C5aR1^−/−^ mice using specific C5aR1 primers (5′-GACCCATAGATAACAGCAGCTTTG-3′; 5′-CCCTCGAGCTAGAGGTACCCTAG-3′; 5′-ACCCACCATCAGCCGCAGGATGGC-3′). Mice were allowed 7 days to acclimatize to the new housing conditions before the commencement of all experiments. All experimental procedures were approved by the University of Queensland Animal Ethics Committee and were conducted in accordance with the Queensland Government Animal Research Act 2001, associated Animal Care and Protection Regulations (2002 and 2008), and the Australian Code of Practice for the Care and Use of Animals for Scientific Purposes, 8th Edition (National Health and Medical Research Council, 2013).

### Experimental Groups and Protocol

Separate cohorts of animals were used to determine 1) the involvement of C5aR1 signaling in the stress response using a group of WT mice, 2) the corticosterone response and additional metabolic and endocrine parameters detecting the effects of acute stress exposure between WT and C5aR1^−/−^ mice, 3) subchronic stress–related behavioral changes between WT and C5aR1^−/−^ mice, and 4) the effects of chronic stress on C5a-C5aR1 signaling in addition to gene and protein expression of stress and inflammatory-related markers between WT and C5aR1^−/−^ mice. Further details are provided in the Supplement.

### C5a Enzyme-Linked Immunosorbent Assay

Plasma C5a levels were determined using the DuoSet mouse complement component C5a enzyme-linked immunosorbent assay (ELISA) according to the manufacturer’s instructions (DY2150, R&D Systems). Levels of C5a in plasma samples were determined from a standard curve ranging from 15.6 to 500 pg/mL.

### Plasma Corticosterone Assay

Plasma corticosterone concentrations were determined using an in-house radioimmunoassay using a highly specific ovine antirat corticosterone polyclonal antibody (Sapphire Bioscience Pty Ltd.) and tritiated (1, 2, 6, 7-^3^H) corticosterone tracer as previously described by Spiers *et al.* (28). Concentrations of unknown samples were determined from a standard curve and corrected for dilution. The intra- and interassay coefficients of variation for the acute stress experiment were 4.8% and 3.6%, respectively. For the chronic stress experiment, the intra-assay coefficient of variation was 6.2%.

### Plasma ACTH and Insulin Assay

A commercially available ELISA kit was used for the quantitative measurement of ACTH in EDTA plasma (Immunotag Mouse ACTH [corticotropin] ELISA kit) according to the manufacturer’s instructions (G-Biosciences). Plasma insulin concentrations were determined using the Ultra-Sensitive Mouse Insulin ELISA Kit (Crystal Chem) according to the manufacturer’s instructions.

### Metabolic Profiling

WT and C5aR1^−/−^ mice were subjected to intraperitoneal glucose and insulin tolerance tests and the ACTH stimulation test. Further details are provided in the Supplement.

### Real-Time Polymerase Chain Reaction

The messenger RNA (mRNA) transcript levels of inflammation- and stress-related genes in the hippocampus and adrenal glands of WT and C5aR1^−/−^ mice were measured using real-time polymerase chain reaction. Further details are provided in the Supplement.

### Immunofluorescence

Immunofluorescence was used for the estimation of C5aR1 expression in astrocytes and microglia within the CA3 region of the hippocampus. Further details are provided in the Supplement.

### Cytokine and Chemokine Measurement

Cytokine and chemokine levels in the hippocampus of WT and C5aR1^−/−^ mice were quantified using the BioLegend LEGENDplex Mouse Inflammation panel kit (BioLegend). Further details are provided in the Supplement.

### Statistical Analysis

Statistical analyses were performed using GraphPad Prism (version 10.1.1; GraphPad Software Inc.). Stress-induced changes in mRNA expression and plasma C5a were compared using 1-way analysis of variance with Fisher’s post-test or Kruskal-Wallis test with Dunn’s multiple comparisons when group standard deviations were significantly different. Stress-induced hormonal and metabolic responses, body weight gain, and locomotor activity in C5aR1^−/−^ mice were compared with WT mice using 2-way repeated-measures analysis of variance with Bonferroni’s multiple comparisons test. Tissue mRNA and cytokine levels in WT and C5aR1^−/−^ mice were compared using 2-way analysis of variance followed by Fisher’s post-test. The Grubbs’ test was applied to identify significant outliers (α < 0.05). Data are presented as bar graphs with all superimposed individual data points, and differences were considered significant when *p* < .05.

## Results

### Stress Induces Elevated Circulating C5a Concentrations While Decreasing C5aR1 Expression in the Hippocampus and Adrenal Gland

**Figure 1:**
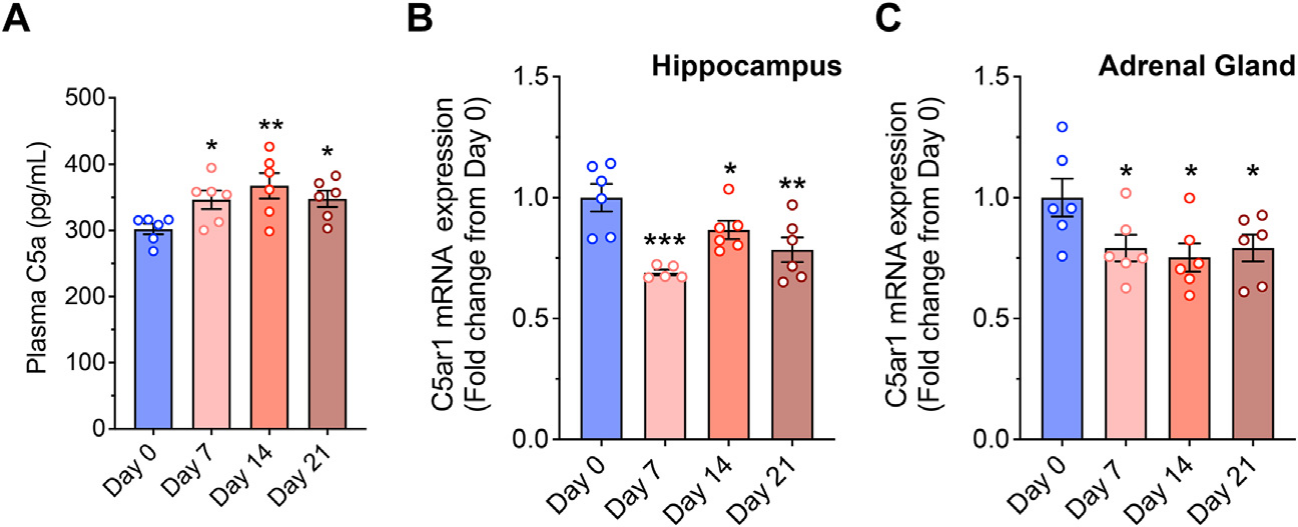
**(A)** Plasma C5a levels and **(B)** hippocampal and **(C)** adrenal gland *C5ar1* mRNA expression from wild-type mice following 7, 14, and 21 days of 2-hour restraint stress compared with nonstressed (day 0) control mice (*n* = 6 per group). Data are expressed as mean ± SEM, ∗*p* < .05, ∗∗*p* < .01, and ∗∗∗*p* < .001. C5ar1, C5a receptor 1; mRNA, messenger RNA.

We have previously observed C5aR1 expression in the CA3 hippocampal subfield, a region critically important for glucocorticoid-mediated negative feedback of the HPA axis (29). Therefore, we investigated whether C5a and C5aR1 expression was sensitive to stress exposure, an indicator that C5aR1 signaling may play a greater role in the stress response. Using a chronic restraint stress paradigm, we found that circulating levels of the complement activation fragment C5a were significantly elevated in WT mice by 15%, 22%, and 15% following 7, 14, and 21 days of restraint stress, respectively, compared with the baseline levels on day 0 (Figure 1A). Moreover, mRNA expression of the receptor *C5ar1* was reduced by approximately 0.3-, 0.13-, and 0.22-fold in the hippocampus following 7, 14, and 21 days of restraint stress, respectively, compared with nonstressed control mice (Figure 1B). We also found that *C5ar1* mRNA expression was reduced by 0.21-, 0.25-, and 0.21-fold in the adrenal gland following 7, 14, and 21 days of restraint stress, respectively (Figure 1C). Additionally, we found that mRNA levels of the central component of complement cascade *C3* and its receptor *C3ar* were increased by 7.3- and 6.2-fold in the hippocampus following 21 days of restraint stress, respectively, compared with nonstressed control mice (Figure S1). Interestingly, the mRNA levels of *C3* and *C3ar* were further increased by 2.1- and 3.6-fold in the hippocampus of mice lacking C5aR1 following 21 days of restraint stress, respectively, suggesting the occurrence of a compensatory mechanism in these mice (Figure S1). These findings demonstrate that exposure to stress leads to complement-mediated C5a production and altered adrenal and hippocampal C5aR1 expression.

### C5aR1 Signaling Modulates the Acute Stress Response and Regulates Adrenal ACTH Sensitivity

**Figure 2:**
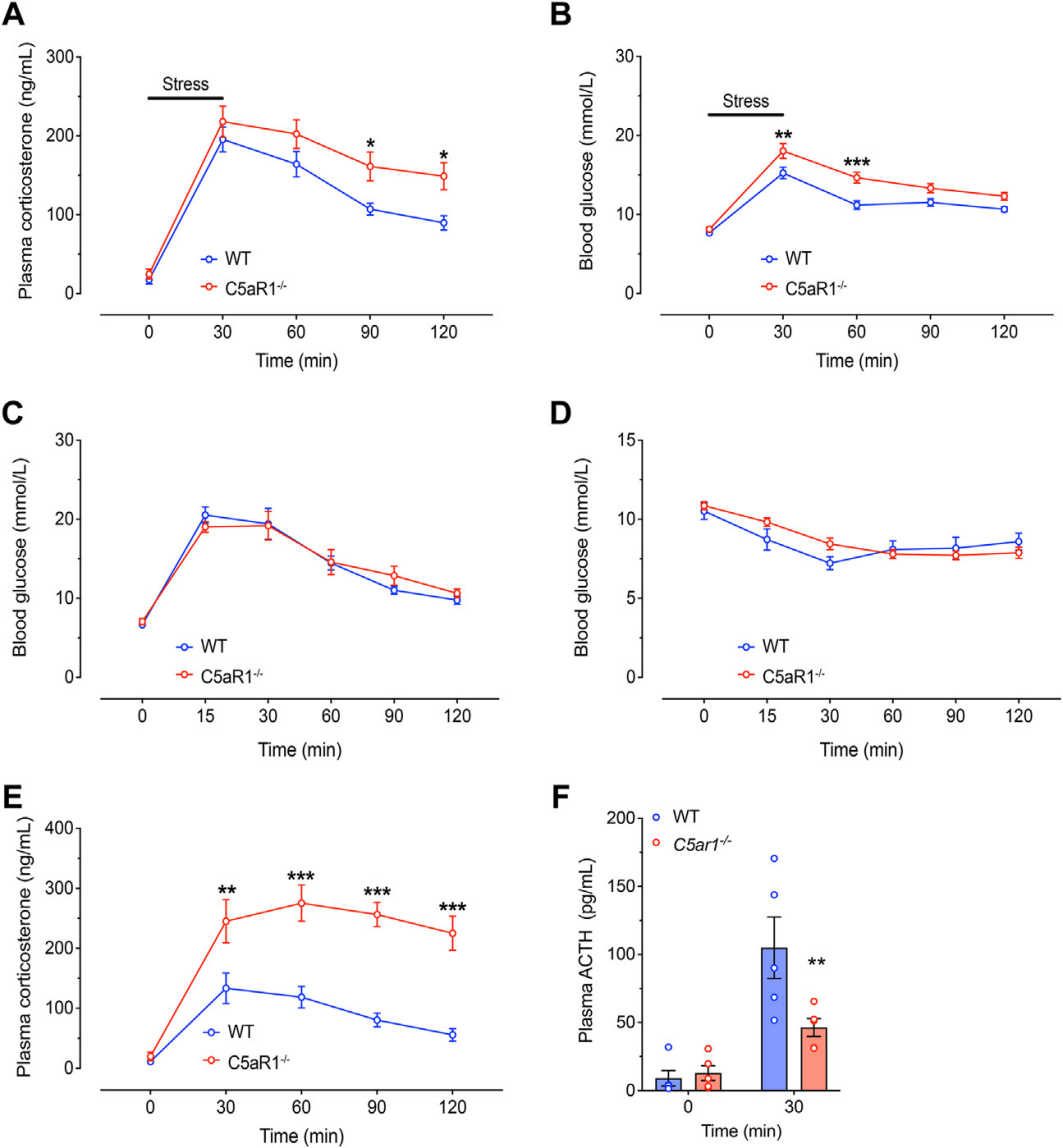
**(A)** Plasma corticosterone concentrations and **(B)** blood glucose levels from WT and C5aR1^−/−^ mice following a single episode of 30-minute restraint stress (*n* = 9–10 per group). Plasma corticosterone and blood glucose concentrations were determined from serial blood samples collected at 0, 30, 60, 90, and 120 minutes. **(C**, **D)** Blood glucose concentrations following **(C)** glucose tolerance test and **(D)** insulin tolerance test from WT and C5aR1^−/−^ mice (*n* = 5–6 per group). **(E)** Plasma corticosterone concentrations following synacthen stimulation from WT and C5aR1^−/−^ mice (*n* = 5–6 per group). **(F)** Plasma ACTH concentrations from WT and C5aR1^−/−^ mice following a single episode of 30-minute restraint stress (*n* = 5 per group). Data are expressed as mean ± SEM. ∗*p* < .05, ∗∗*p* < .01, and ∗∗∗*p* < .001. ACTH, adrenocorticotropic hormone; C5aR1, C5a receptor 1; WT, wild-type.

To identify whether C5aR1 signaling directly alters HPA axis output, circulating levels of the stress hormone corticosterone were measured in WT and C5aR1^−^^/^^−^ mice following a 30-minute period of restraint stress. No differences were observed in peak circulating corticosterone levels at 30 minutes poststress between both groups. However, C5aR1^−/−^ mice demonstrated an impaired ability to recover following stress exposure, exhibiting 51% and 66% higher corticosterone concentrations at 90 and 120 minutes after the onset of stress, respectively (Figure 2A). During a stress response, glucocorticoids and several other hormones such as catecholamines also promoted the mobilization of glucose to facilitate adenosine triphosphate production. Following 30 minutes of restraint stress, C5aR1^−/−^ mice had 18% and 31% higher blood glucose concentrations at 30 and 60 minutes, respectively, poststress compared with WT control mice (Figure 2B). The observed elevation in blood glucose levels could indicate a dysregulated ability to respond to hyperglycemia. Therefore, we tested this using the intraperitoneal glucose and insulin tolerance tests. Interestingly, there were no differences in blood glucose levels between WT and C5aR1^−/−^ mice following either the glucose or insulin tolerance tests (Figure 2C, D). Furthermore, the basal level of insulin was not different between the 2 genotypes (Figure S2A). To identify whether the elevations in corticosterone were generated centrally or via a peripheral response, we also assessed the sensitivity of the adrenal gland in producing corticosterone by performing an ACTH stimulation test. We found that C5aR1^−/−^ mice had highly potentiated adrenal sensitivity to ACTH, with 88%, 132%, 219%, and 305% higher corticosterone outputs than WT control mice at 30, 60, 90, and 120 minutes, respectively, after ACTH administration (Figure 2E). Moreover, although there were no differences in basal ACTH levels, the loss of C5aR1 signaling attenuated the stress-induced ACTH levels to approximately 44% of the WT control mice (Figure 2F).

### Genetic Deletion of C5aR1 Disrupts Stress-Induced Locomotion, Glucose/Insulin Homeostasis, and Glucocorticoid Receptor Expression

**Figure 3:**
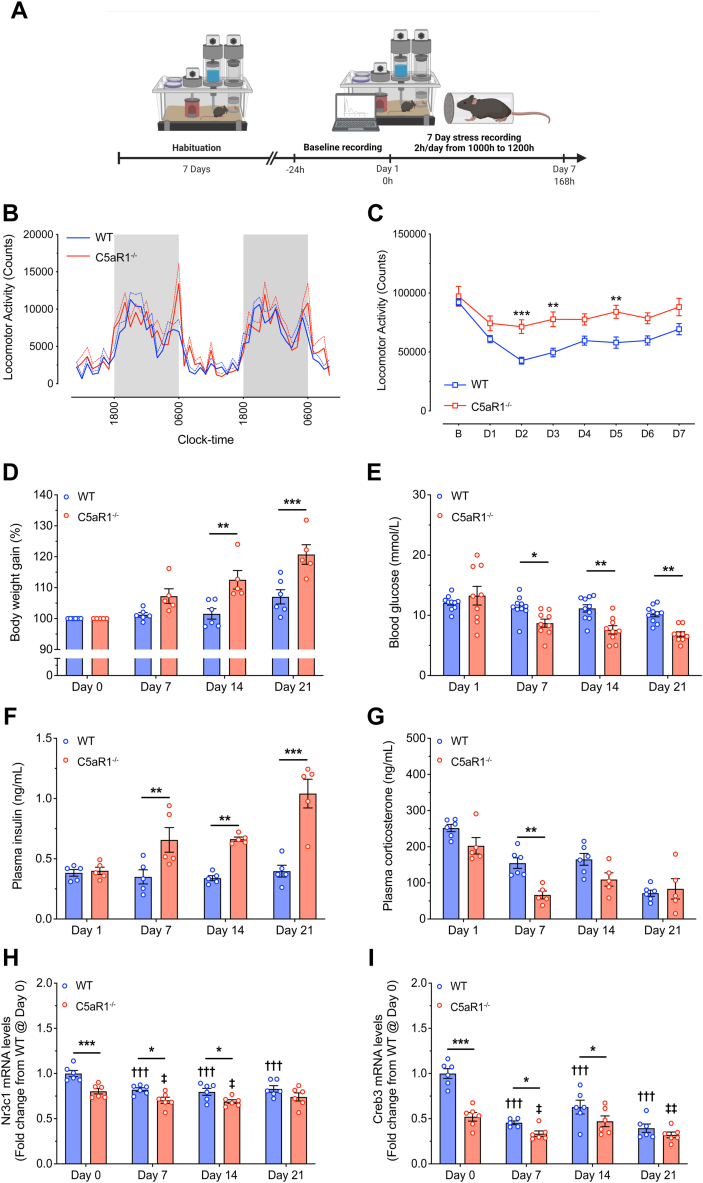
**(A)** Schematic recording timeline for the PhenoMaster experiment, **(B)** line graph demonstrating 24-hour continuous recording of total locomotor activity under basal conditions, and **(C)** total locomotor activity from baseline recording (“B”) and following daily restraint stress for 7 days from WT and C5aR1^−/−^ mice (*n* = 7–8 per group). **(D)** Body weight gain, **(E)** blood glucose, **(F)** plasma insulin, and **(G)** plasma corticosterone concentrations from WT and C5aR1^−/−^ mice following 7, 14, and 21 days of 2-hour restraint stress compared with nonstressed control mice (*n* = 5–10 per group). **(H)** Hippocampal glucocorticoid receptor *Nr3c1* and **(I)***Creb3* mRNA expression from WT and C5aR1^−/−^ mice following 7, 14, and 21 days of 2-hour restraint stress compared with nonstressed control mice (*n* = 6 per group). Data are expressed as mean ± SEM with ∗*p* < .05, ∗∗*p* < .01, and ∗∗∗*p* < .001 when comparing between strains and ^†/‡^*p* < .05, ^††/‡‡^*p* < .01, and ^†††/‡‡‡^*p* < .001 when comparing with respective nonstressed (day 0) values. C5aR1, C5a receptor 1; mRNA, messenger RNA; WT, wild-type.

The observation of stress-induced changes in C5a and C5aR1 expression and key acute changes in HPA axis function in the absence of C5aR1 led us to investigate how loss of C5aR1 would affect the response to chronic stress. Initially, we used an automated behavioral phenotyping system (TSE PhenoMaster) to monitor broad stress-induced changes in basal activity extending beyond the acute response into the subchronic domain of stress using daily exposure to restraint stress for 7 days (Figure 3A). No differences were observed in baseline levels of locomotor activity prior to any stress exposure (Figure 3B). However, exposure to a 2 hours of restraint stress daily predictably reduced activity in both WT and C5aR1^−/−^ mice by 34% and 24%, respectively (Figure 3C). Furthermore, this reduction was generally attenuated in C5aR1^−/−^ mice, with approximately 40% higher locomotor activity levels than WT control mice from day 2 of restraint stress exposure (Figure 3C).

We further evaluated the impact of C5aR1 deficiency with a well-established chronic model of restraint stress using daily 2-hour stress exposure for up to 21 days, a period known to disrupt multiple aspects of normal physiology and induce depressive-like behaviors (30). Chronic stress is known to attenuate the weight gain typically observed in young adult mice. We found that WT mice exposed to daily restraint stress exhibited largely abrogated weight gain over the treatment period (Figure 3D). Strikingly, this stress-induced weight gain suppression was not observed in C5aR1^−/−^ mice (Figure 3D). Following 14 and 21 days of restraint stress exposure, C5aR1^−/−^ mice demonstrated 11% and 13.7% greater body weight gain, respectively, compared with the WT control mice (Figure 3D). To assess whether changes in body weight gain were being driven by altered glucose homeostasis, we next measured circulating glucose, insulin, and corticosterone levels. Reductions of approximately 24%, 32%, and 33% were observed in glucose levels of C5aR1^−/−^ mice at 7, 14, and 21 days of restraint stress exposure, respectively, compared with WT control mice (Figure 3E). Next, we measured insulin levels because insulin is the primary hormone drives glucose uptake. Stress treatment had no discernible effect on insulin in WT mice over the duration of the experiment. However, in C5aR1^−/−^ mice, stress significantly increased insulin by 88%, 96%, and 162% at 7, 14, and 21 days of treatment, respectively, compared with WT control mice (Figure 3F). Furthermore, a 57% reduction in stress-induced corticosterone was observed in C5aR1^−/−^ mice at 7 days (Figure 3G), coinciding with the reduced levels of glucose (Figure 3E) and attenuated stress-induced relative adrenal weight (Figure S2B). Assessment of adrenal inflammatory gene expression also showed generalized reductions in proinflammatory cytokine expression (*Tnf*, *Il1b*, and *Il6*) initially, with no further differences being observed following stress exposure (Figure S2C–E).

The hippocampus is a brain region that is extremely susceptible to fluctuations in glucocorticoid signaling. Mice lacking C5aR1 showed reduced mRNA expression of the glucocorticoid receptor (GR) *Nr3c1* across all time points measured (Figure 3H). Reductions in GR expression is expected during chronic stress exposure due to the well-characterized hippocampal negative feedback response to glucocorticoids (31,32). However, C5aR1^−/−^ mice showed lower baseline and sustained reductions in hippocampal GR expression over the course of the experiment despite exhibiting relatively similar glucocorticoid concentrations. This suggests that C5a-C5aR1 signaling may modulate functional sensitivity of the GR, an attribute regulated in part by the endoplasmic reticulum stress protein and GR transcriptional regulator CREB3. Therefore, we examined hippocampal *Creb3* mRNA expression and identified that its expression was markedly decreased in nonstressed C5aR1^−/−^ mice (Figure 3I), and this difference between WT and C5aR1^−/−^ mice remained up until 14 days of stress exposure. Moreover, both genotypes showed reduced expression of hippocampal *Creb3* in response to chronic stress treatment (Figure 3I).

### C5a-C5aR1 Signaling Modulates Expression of Hippocampal Microglia During Chronic Stress

**Figure 4:**
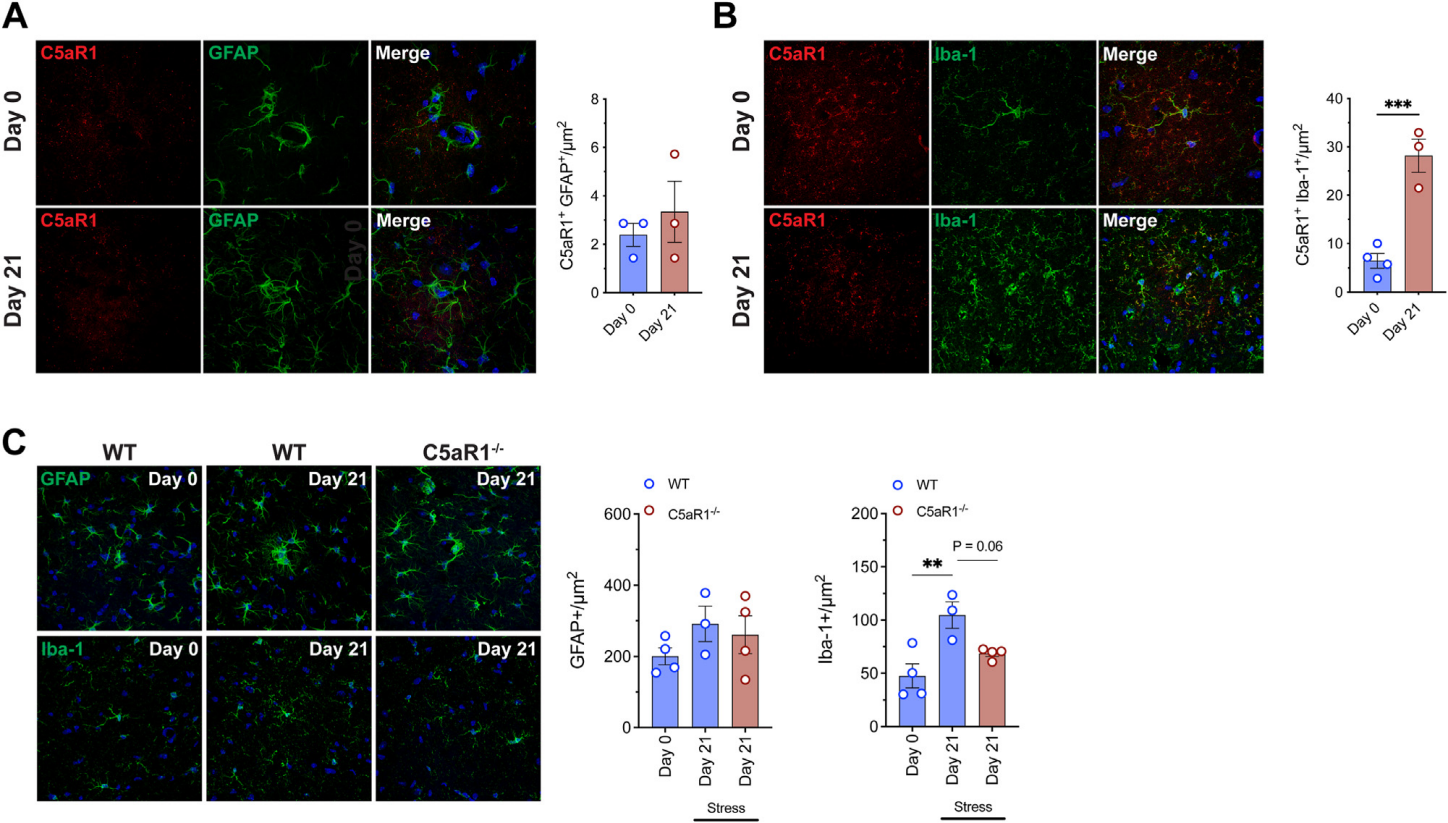
**(A)** Double immunolabeling of C5aR1 (red) with astrocytes (GFAP; green) and quantification in the CA3 region of the hippocampus of WT mice following 21 days of 2-hour restraint stress compared with nonstressed (day 0) control mice (*n* = 3 per group). **(B)** Double immunolabeling of C5aR1 (red) with microglia/macrophages (Iba-1; green) and quantification in the CA3 region of the hippocampus of WT mice following 21 days of 2-hour restraint stress compared with nonstressed (day 0) control mice (*n* = 3–4 per group). **(C)** GFAP-positive astrocytes and Iba-1 positive microglia/macrophages in the CA3 region of the hippocampus of WT and C5aR1^−/−^ mice at 21 days of restraint stress compared with nonstressed control mice (*n* = 3–4 per group). Data are expressed as mean ± SEM. ∗∗*p* < .01, and ∗∗∗*p* < .001. Scale bars for all panels = 25 μm. C5aR1, C5a receptor 1; WT, wild-type.

To further investigate the role of C5a-C5aR1 signaling during chronic stress exposure, we examined the expression of hippocampal glial markers because these cells largely mediate neurological immunity and likely use complement signaling to facilitate the innate immune response (33). Initially, the coexpression of C5aR1 with either GFAP-positive astrocytes or Iba-1–positive microglia were examined in the hippocampal CA3 region using immunofluorescence in WT mice. While no significant difference was observed in the number of C5aR1-expressing astrocytes under stress, there was a significant increase in the number of C5aR1-expressing microglia following 21 days of restraint stress in WT mice (Figure 4A, B). Additionally, no significant alteration was observed in the number of GFAP-positive astrocytes in the hippocampus of WT or C5aR1^−/−^ mice when subjected to 21 days of restraint stress (Figure 4C). However, there was a significant increase in the number of Iba-1 positive microglia in WT mice in response to 21 days of restraint stress, which was attenuated in C5aR1^−/−^ mice (Figure 4C). Intriguingly, upon examining the protein expression levels of cytokines and chemokines in the hippocampus of WT mice following 21 days of restraint stress, we observed a decrease in IL-23, IL-6, interferon beta, and MCP-1 compared with nonstressed WT control mice (Figure S3). However, the decrease in IL-23 was the only cytokine attenuated in stressed C5aR1^−/−^ mice compared with stressed WT mice (Figure S3A).

## Discussion

**Figure 5:**
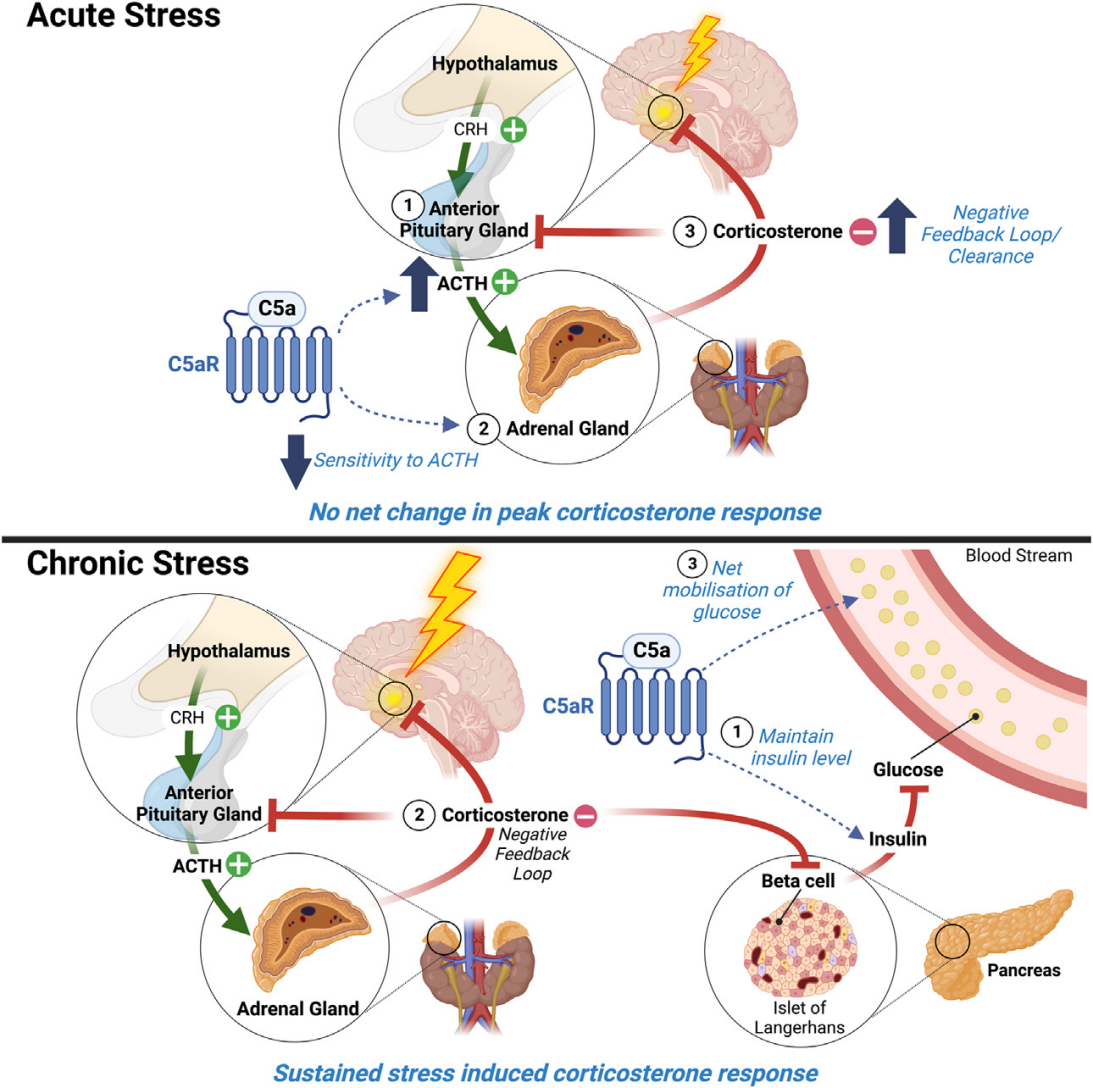
Schematic summary of C5a-C5aR1 signaling on hypothalamic-pituitary-adrenal axis. (Top panel) Under acute stress, C5a-C5aR1 signaling enhances pituitary gland (1) output of ACTH while simultaneously reducing adrenal gland (2) sensitivity to ACTH. This results in no net change in the peak response of corticosterone (3) to an acute stressor. However, C5a-C5aR1 signaling alters the rate of corticosterone recovery to baseline levels by influencing increased negative feedback/clearance mechanisms. (Bottom panel) In conditions of chronic stress, C5a-C5aR1 signaling helps to maintain basal insulin levels (1) and sustains the corticosterone response (2) to stress by delaying habituation. This results in a net mobilization of blood glucose (3). ACTH, adrenocorticotropic hormone; C5aR1, C5a receptor 1; CRH, corticotropin-releasing hormone.

It has been suggested that stress-induced dysregulation of immune function is a major factor contributing to neuropsychiatric conditions such as depression (3, 4, 5, 6). Chronic stress is particularly disadvantageous for neuronal homeostasis due to the prolonged glucocorticoid exposure occurring via long-term activation of the HPA axis (34). However, the relationship between chronic stress and inflammation in the brain is multifaceted, with predictable stressors generally favoring immunosuppression while unpredictable models promote targeted levels of inflammation (35,36). In this study, we demonstrated that complement C5a-C5aR1 signaling exerts important and complex modulatory activities over the HPA axis during acute and chronic stress exposure (Figure 5). The expressions of C5a and C5aR1 in circulation and stress-sensitive regions of the brain were altered following stress, suggesting that complement activation could play a key role in modulating the stress response. In mice lacking C5aR1, the response to acute stress showed reduced ACTH output with compensatory facilitation of ACTH sensitivity while prolonged stress exhibited a degree of glucocorticoid insufficiency. Initially, this facilitated adrenal sensitivity may be the result of a reduced inflammatory phenotype in the adrenal gland (Figure S2C–E). Francis *et al.* (24) demonstrated expression of functional C5aR1 in the anterior pituitary gland and suggested that this promoted ACTH release leading to anti-inflammatory glucocorticoid production. This is consistent with our data showing reduced ACTH output in mice lacking C5a-C5aR1 signaling. During the transition from acute to chronic stress, C5aR1 knockout mice displayed ameliorated reductions in locomotion and greater relative weight gain during chronic stress treatment, suggesting a degree of resilience to stress in this early period. Conversely, prolonged stress exposure in mice lacking C5aR1 promoted significant hyperinsulinemia and impaired glucose mobilization, an indicator of metabolic dysfunction observed prior to insulin resistance in the early development of diabetes (37, 38, 39). In the hippocampus, expression of GR and Creb3, a modulator of GR sensitivity, were consistently reduced in C5aR1 knockout mice, suggesting an overall genotype-specific alteration in sensitivity to glucocorticoids, a phenotype also observed in patients with diabetes (40). A complex relationship exists between GR and Creb3, with Creb3 acting as both a GR cofactor and a transcription factor capable of interacting with glucocorticoid response elements to promote transcriptional repression of GR activity via epigenetic histone modification (41,42). This relationship may begin to explain how loss of C5a-C5aR1 signaling differentially affects the response to stress over acute and chronic time domains. However, further studies are needed to understand how C5a-C5aR1 signaling mechanistically interacts with HPA axis mediators such as GR in the hippocampus, particularly given the important role of this receptor in mediating negative feedback.

In addition to mediating glucose homeostasis, the anti-inflammatory effects of glucocorticoids and insulin have been well documented in peripheral tissues. While the effects of insulin seem to be predominately anti-inflammatory, in the brain, the actions of glucocorticoids are much less clear and seemingly rely heavily on the type of stress and duration of exposure. The predictable model of stress used in the current study has been characterized as significantly immunosuppressive in comparison to unpredictable models in which levels of corticosterone tend to remain consistently elevated (43). However, both models of stress have been observed to reduce neuronal proliferation and survival in the hippocampus in addition to displaying different degrees of glial remodeling (44). Interestingly, previous studies have demonstrated the involvement of the classical complement pathway in synaptic pruning, as evidenced by defects in synapse elimination in postnatal mice with C1q or C3 deficiency (45). It would be interesting to investigate whether C5a-C5aR1 signaling further modulates synaptic plasticity under stress conditions. In the current study, there was an increase in microglia/macrophages expressing C5aR1 in the hippocampus of chronically stressed WT mice that was largely driven by increased Iba-1 expression. Loss of C5aR1 signaling reduced the number of Iba-1 positive microglia/macrophages, indicating that there is a strong interplay between C5a-C5aR1 signaling in microglia/macrophages during the later stages of the stress response. Moreover, there was a significant increase in the number of Iba-1 positive microglia/macrophages in the CA3 region of the hippocampus following 21 days of restraint stress. This finding is consistent with previous published literature that used a similar stressor and reported elevated Iba-1–positive cell counts in stress-responsive regions including the hippocampus, medial prefrontal cortex, and nucleus accumbens (46). These results suggest that microglia/macrophages may play a key role in the development of neuroinflammation induced by chronic stress. Furthermore, another study demonstrated that after 21 days of chronic unpredictable stress, there was a notable increase in the Iba-1-positive cells expressing another complement receptor, C3aR, in the prefrontal cortex (47). The same study also found heightened C3 expression in the prefrontal cortex of individuals with depression and showed that mice lacking C3 exhibited resilience to chronic stress–induced depressive-like behavior. Here, we have shown similar results. Compensatory C3-C3aR signaling was observed alongside increased Iba-1–positive cells in the hippocampus in chronically stressed WT mice. However, because C3 signals upstream from C5, it seems that this compensatory increase in C3 signaling does not drive excessive microglial activation in the C5aR1 knockout mice. Further studies should elucidate the importance of C5aR1 signaling in the neuroinflammatory aspects of chronic stress, particularly because higher levels of C5a have been found in the cerebrospinal fluid of patients with major depressive disorder, a condition generally exacerbated by stress (48,49). These findings emphasize the significance of complement components in modulating stress-related disorders. Moreover, anticomplement therapy may be a useful clinical tool in the treatment of these disorders, particularly when inflammation is observed, offering a safe nonsteroidal anti-inflammatory option for use individually or in combination with currently available treatments. Although the role of complement signaling in neurological disorders is not yet fully understood, the recent development of small-molecule complement inhibitors capable of crossing the blood-brain barrier (50,51) provides new hope that these may offer new therapeutic strategies for neurological disorders in a clinical setting. It should be noted that only male mice were used in this study, highlighting the need for further research to determine whether C5a-C5aR1 signaling has similar modulatory effects on the stress system in female mice. Additionally, the use of global C5aR1 knockout mice presents challenges in isolating central and peripheral components affected by stress. Future studies utilizing targeted and conditional knockouts would provide more nuanced insights into the physiological responses following stress exposure.

### Conclusions

This study provides evidence of a novel C5a-C5aR1 modulatory mechanism that, when disrupted, precipitates glucose/insulin dyshomeostasis and promotes a proinflammatory phenotype under conditions of chronic stress. We showed differential modulation of the stress response by C5aR1 at multiple levels of the HPA axis, with the downstream effects of stress exposure depending heavily on stress duration. Overall, this highlights the C5a-C5aR1 signaling axis as an important factor in maintaining stress resilience under conditions of chronic stress.

## Supplementary Material

Key Resources Table
