# Supplementary material for: Complement C5a Receptor Signaling Alters Stress Responsiveness and Modulates Microglia Following Chronic Stress Exposure
Source: Biol Psychiatry Glob Open Sci. 2024 Mar 7;4(3):100306. doi: 10.1016/j.bpsgos.2024.100306 (PMC11019103; doi:10.1016/j.bpsgos.2024.100306)
Supplement: Supplement [file mmc2.pdf]

SUPPLEMENTARY INFORMATION

Complement C5a Receptor Signaling Alters Stress Responsiveness and Modulates Microglia Following Chronic Stress Exposure

Chen *et al.*

Supplementary Materials and Methods

Experimental groups and protocol

Cohort One

One week prior to experimental manipulation, a group of WT mice were habituated to human handling for 10 min each day. On each experimental day, mice were transported to an experimental room one hour before commencement of stress treatment. Food and water were removed during this habituation and treatment period. Following the habituation protocol, WT mice (n = 6/group) were randomly allocated to groups of non-stressed or 7, 14, or 21 days of 2-hour restraint stress from 1000h to 1200h. At the end of each allocated treatment, mice were anaesthetised with intraperitoneal injection of zolazepam (50 mg/kg, Zoletil; Lyppard) and xylazine (10 mg/kg, Xylazil; Lyppard). A terminal blood sample was then collected in 0.01 M EDTA (anticoagulant) and 0.01 M nafamostat mesilate (FUT-175; inhibitor of C3/C5 convertases) followed by centrifugation at 1000 x *g* for 10 min at 4°C and the resulting plasma was stored for the C5a ELISA. Fresh brains were rapidly removed and the whole hippocampus was excised in addition to the adrenal glands and were stored at -80°C for later relative gene expression analysis.

Cohort Two

Following the same habituation protocol, WT and C5aR1$^{-/-}$ mice were subsequently restrained using cylindrical metal restrainers (3 x 7 cm) equipped with nasal ventilation holes for 30 minutes from 1000h to 1030h and blood samples were collected via the tail using heparinised capillary tubes at 0 (baseline), 30, 60, 90, and 120 minutes for repeated blood glucose and plasma corticosterone measurements (n = 9-10 per group). Blood glucose was determined using a standard glucometer (FreeStyle Optium Neo, Abbott) and whole blood samples were subsequently centrifuged at 1000 x *g* for 10 min, after which supernatant plasma was collected

and stored at -80°C for later determination of corticosterone concentrations. For plasma ACTH determination, whole blood samples were collected into EDTA-coated capillary tubes at 0 (baseline) and 30 (cessation of stress) minutes followed by centrifugation at 1000 x *g* for 10 min, after which aprotinin (50KIU/µL) was added to the supernatant plasma and stored at -80°C for later determination of plasma ACTH. Separate groups of WT and C5aR1$^{-/-}$ mice (n = 5-6) were used for glucose/insulin tolerance and ACTH stimulation tests.

Cohort Three

Continuous assessment of basal behaviours and physiological parameters including locomotor activity following stress exposure was determined in WT and C5aR1$^{-/-}$ mice (n = 8) utilising the TSE PhenoMaster as described previously (1). One week prior to experimental manipulation, WT and C5aR1$^{-/-}$ male mice were relocated to the housing cages within the PhenoMaster system and habituated to human handling for 10 min each day. Mice were housed individually with free access to food and water with cage temperatures tightly regulated at 24.5 ± 0.1°C in cages equipped with an infrared light beam frame (ActiMot) for real time measurement of locomotor activity, defined as the total number of infrared beam breaks in the X and Y-axis (counts). The baseline levels for locomotor activity prior to the application of restraint stress was established for 24 hours within the automated TSE PhenoMaster system. All mice were subsequently subjected to 2 hours of restraint stress from 1000h to 1200h each day for 7 days and immediately following each stress cessation, mice were monitored for locomotor activity from Day 1 to 7.

Cohort Four

Following the same habituation protocol as Cohort One, WT and C5aR1$^{-/-}$ mice (n = 5-10) were randomly allocated to groups of non-stressed or 7, 14, or 21 days of 2-hour restraint stress from

1000h to 1200h. During the treatment period, body weights were recorded for repeated body weight measurements. Blood samples were collected via the tail at the end of each stress treatment for repeated blood glucose, insulin, and plasma corticosterone measurements. After each session of restraint stress, mice were returned to their home environment with free access to food and water.

Similarly, a separate group of WT and C5aR1$^{-/-}$ mice (n = 6) were allocated to groups of non-stressed or 7, 14, or 21 days of 2-hour restraint stress from 1000h to 1200h. At the end of each allocated treatment, mice were anaesthetised with intraperitoneal injection of zolazepam and xylazine. Whole hippocampus was excised and stored for later relative gene and protein expression analysis. Additionally, the absolute weight of adrenal glands was weighed, normalised to individual body weight, and expressed as mg/100 g body weight to enable comparisons between treatment groups and strains. The adrenal glands were then stored at -80°C for later relative gene expression analysis. For immunohistochemical analysis, WT and C5aR1$^{-/-}$ mice (n = 3 – 4) were transcardially perfused with 2% sodium nitrite in 0.1 M phosphate buffer (pH = 7.4) followed by 4% paraformaldehyde (Sigma, St. Louis, MO, USA) in 0.1 M phosphate buffer (pH=7.4). Whole brains were then collected and post-fixed in 4% paraformaldehyde for 2 hours at 4°C followed by submersion in 15% and 30% sucrose. The brains were subsequently embedded in Tissue-Tek® optimal cutting temperature compound (Sakura Finetek, Torrance, CA), and snap frozen in liquid nitrogen. Serial cryosections (16 µm) were collected on SuperFrost® plus slides (Menzel Gläser, Braunschweig) for immunohistochemical staining.

Metabolic profiling

WT and C5aR1$^{-/-}$ mice were habituated to human handling for 10 min each day for one week. Mice were fasted for 16 hr overnight (maintaining free access to water) then subjected to the intraperitoneal glucose tolerance test with 2 g/kg body weight of glucose in normal saline. Tail blood was used to measure glucose concentration at 0, 15, 30, 60, 90, and 120 minutes. A week after the glucose tolerance test, intraperitoneal insulin tolerance tests (0.5 IU/kg body weight insulin in normal saline) were performed in the same group of WT and C5aR1$^{-/-}$ mice following a 6 hour fast. Blood glucose concentrations were determined at 0, 15, 30, 60, 90, and 120 minutes.

For the ACTH stimulation test to examine HPA axis responsiveness, the synthetic glucocorticoid, dexamethasone (50 µg/kg body weight in normal saline) was first administered to supress HPA axis output to prevent biased by potentially higher baseline levels. Mice were then left in an isolated experimental room for 2 hours. Following a blood sample collected at 0 (baseline), a synthetic derivative of ACTH, synacthen, was intraperitoneally administered at a dose of 5 µg/kg body weight (in 0.9% saline). Tail blood samples were then collected at 30, 60, 90, and 120 minutes using heparinised capillary tubes. Whole blood samples were subsequently centrifuged at 1000 x *g* for 10 min, after which supernatant plasma was collected and stored at -80°C for later determination of corticosterone concentrations.

Real-time PCR

Total RNA from the hippocampus and adrenal glands were extracted using the RNeasy® Lipid Tissue Mini Kit (Qiagen, Doncaster, Australia), purified with the Ambion^{TM} TURBO DNA-free^{TM} DNAse treatment (Life Technologies, Mulgrave, Australia), and reversed transcribed into cDNA using the AffinityScript QPCR cDNA Synthesis Kit (Agilent Technologies,

Mulgrave, Australia) according to the manufacturer's instructions. TaqMan gene expression kits (Life Technologies, Mulgrave, Australia) with optimised primers and reporter dye labelled-probes (FAM; 6-carboxyfluorescein) were used to determine gene expression of *C5ar1* (Mm00500292_s1), *Nr3c1* (Mm00433832_m1), *Creb3* (Mm00457268_m1), *Il1b* (Mm00434228_m1), *Il6* (Mm00446190_m1), and *Tnf* (Mm00443258_m1). All expression assays were normalised to the geometric mean of reference genes, glyceraldehyde 3-phosphate dehydrogenase (*Gapdh*; Mm99999915_g1) and beta actin (*Actb*; Mm01205647_g1). The fold change in expression of each target gene was calculated using this formula $2^{-\Delta CT}$ where $\Delta CT = (Ct_{\text{(Target gene)}} - Ct_{\text{(Gapdh and Actb)}})$. Final measures are presented as relative levels of gene expression in WT mice at Day 0 compared with expression in WT and C5aR1$^{-/-}$ mice at Day 7, 14 and 21 of restraint stress.

Immunofluorescence

The brain sections were blocked in PBS containing 3% BSA for 1 hour at room temperature and incubated for 48 hours at 4°C with C5aR1 (rat anti-C5aR1, clone 10/92; 1:100, Bio-Rad Laboratories, Hercules, CA, USA) along with specific cell type markers for astrocyte (mouse anti-GFAP; 1:1000, BD Biosciences, San Diego, CA, USA) or microglia (rabbit anti-Iba1; 1:500,Wako Chemicals, Osaka, Japan). Following the incubation with primary antibodies, sections were washed with PBS and incubated overnight at 4°C with a cocktail of Alexa-conjugated secondary antibodies (1:1000 for Alexa Fluor 555 and 1:600 for Alexa Fluor, Invitrogen, Eugene, OR, USA). All primary and secondary antibodies were diluted in PBS containing 1% BSA. Sections were then washed in PBS and incubated for 10 minutes in 6-diamidino-2-phenylindole (DAPI; Invitrogen, Eugene, OR, USA). All sections were washed in PBS and then mounted with Prolong Gold Anti-Fade (Invitrogen, Eugene, OR, USA). Images of the hippocampus were acquired using a Leica SP8 Point Scanning microscope with

a 40x objective. Random regions (2226 ×2520 µm) within the CA3 region of each section were selected based on DAPI channel viewing. Manual counting of C5aR1-positive astrocytes and microglia, defined as the co-expression with GFAP and Iba-1 immunostaining, respectively was performed. Quantification of GFAP and Iba-1 positive cells were performed on ~25 to 28 brain sections (between Bregma -1.67mm and -2.27mm). All cell counts were expressed as cell/μm^2. Staining procedures and image exposures were all standardized across different groups. The mouse genotype was not disclosed to the researchers until the completion of the study.

Cytokine and Chemokine Measurement

The soluble fraction of protein was extracted from the hippocampus of WT and $C5aR1^{-/-}$ mice for cytometric bead array. All procedures were performed on ice. Tissues samples were homogenised with Precellys 24 Tissue Homogeniser (Bertin Technology, Montigny-le-Bretonneux, France) in lysis buffer (pH 8.0) at a weight-to-volume ratio of 1:7. The lysis buffer contained 120 mM NaCl and 50 mM Tris and was supplemented with 10 µL/mL of protease and phosphatase inhibitor cocktail (Roche, Basel. Switzerland), as described previously (2). Hippocampal homogenates were centrifuged at 12,000 x *g* for 10 minutes at 4°C. Supernatants, consisting of the soluble fraction, were collected stored at -80°C until further use in CBA. Total protein concentration was determined using the BCA assay kit (Pierce, IL, USA). The hippocampal cytokine concentrations, including Granulocyte-macrophage colony-stimulating factor (GM-CSF), interferon-γ (IFN-γ), tumour necrosis factor α (TNFα), monocyte chemoattractant protein 1 (MCP-1), interleukin-1α (IL-1α), IL-1β, IL-6, IL-10, IL-12p70, IL-17A, IL-23, IL-27 and IL-33 were determined using the BioLegend® LEGENDplex™ Mouse Inflammation panel kit (BioLegend, San Diego, CA, USA). All samples were measured in duplicate.

For the CBA assay, wells were filled with 25 µL of bead solution, 25 µL of assay buffer, and 25 µL of standard or 25 µL of 100 µg hippocampal lysates (total volume of 75 µL). Plates were incubated for 2 hours with agitation (500 rpm) at room temperature. Subsequently, 25 µL of Phycoerythrin (PE)-labelled detection antibody was added to each well and incubated for 1 hour with agitation (500 rpm) at room temperature. To detect the secondary antibody, 25 µL of streptavidin-PE was added and incubated for 30 min at room temperature. The median fluorescence intensity of each bead was obtained using a flow cytometry apparatus (BD™ LSR II). Flow cytometry data were processed using LEGENDplex Gognit software (BioLegend, San Diego, CA, USA).

Supplementary Figures

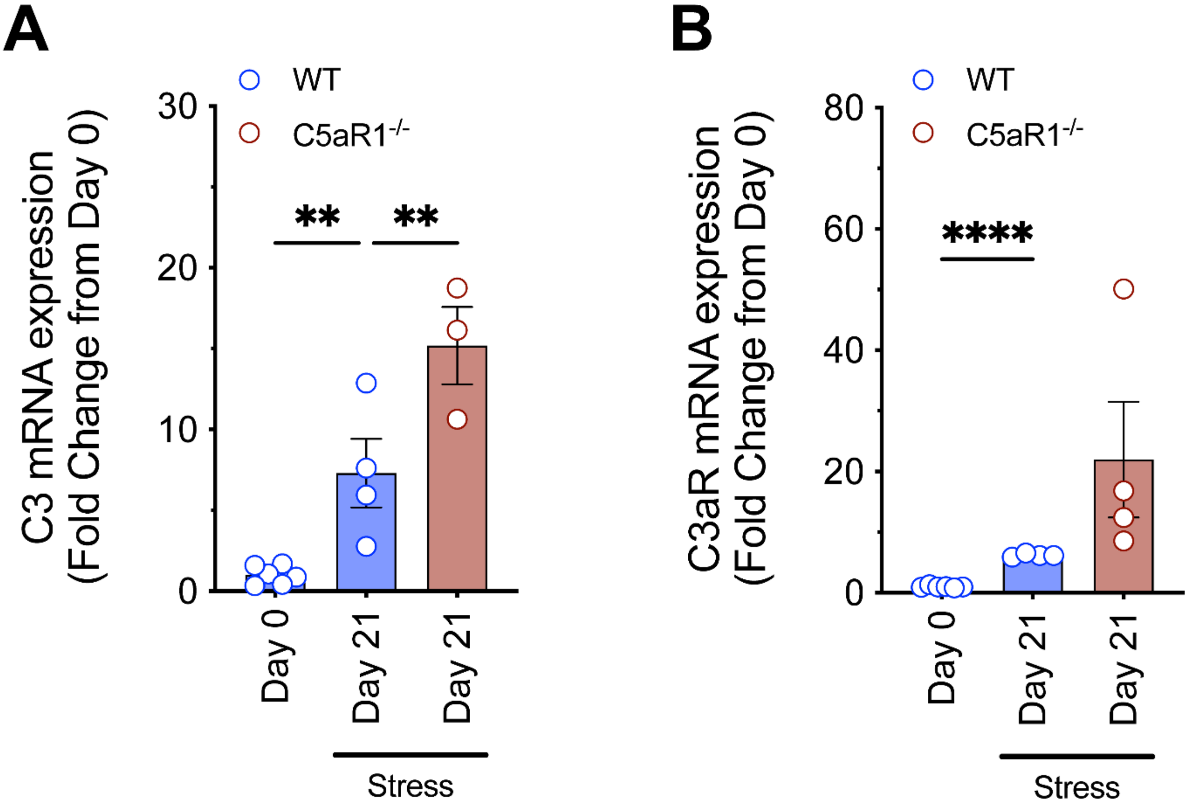

Supplementary Figure 1. Hippocampal complement C3 (**A**) and C3aR (**B**) mRNA expression from wild-type and C5aR1$^{-/-}$ mice following 21 days of 2 h restraint stress compared to non-stressed (Day 0) control (n = 4 – 6 per group). Data are expressed as mean ± SEM, **$p < 0.01$, and **** $p < 0.0001$.

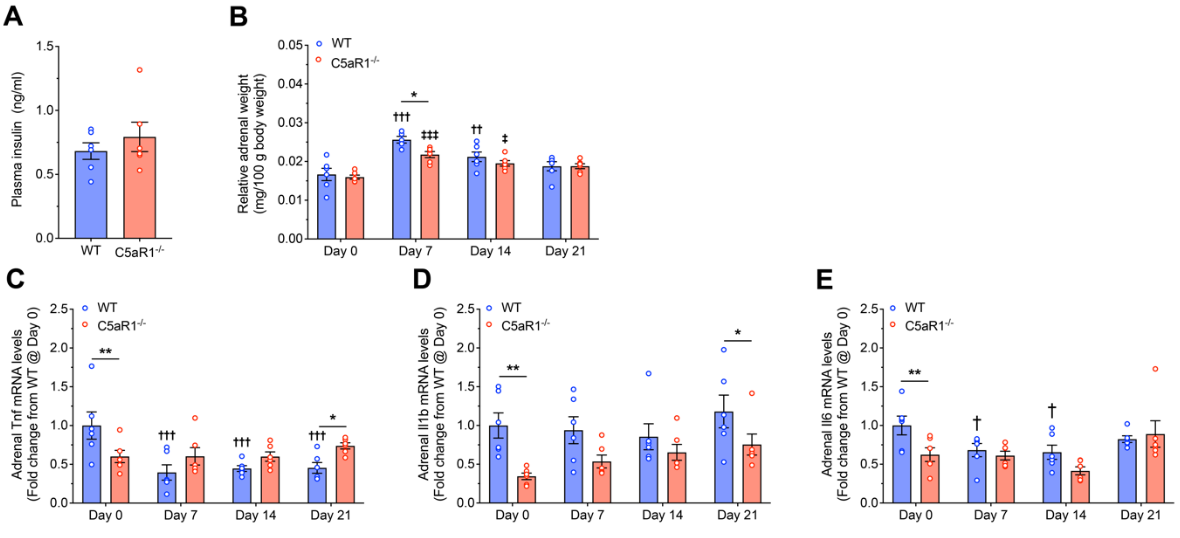

Supplementary Figure 2. (A) Basal plasma insulin concentrations from wild-type (WT) and C5aR1$^{-/-}$ mice (n = 6 per group). **(B)** Relative adrenal weight and adrenal **(C)** tumour necrosis factor (Tnf), **(D)** interleukin 1 beta (Il1b), and **(E)** interleukin 6 (Il6) mRNA expression from WT and C5aR1$^{-/-}$ mice following 7, 14 and 21 days of 2 h restraint stress compared to non-stressed control (n = 6 per group). Data are expressed as mean ± SEM, and *p < 0.05, **p < 0.01 and ***p < 0.001 comparing between strains and $^{†/‡}$p < 0.05, $^{††/‡‡}$p < 0.01, and $^{†††/‡‡‡}$p < 0.001 comparing to respective non-stressed (Day 0) values.

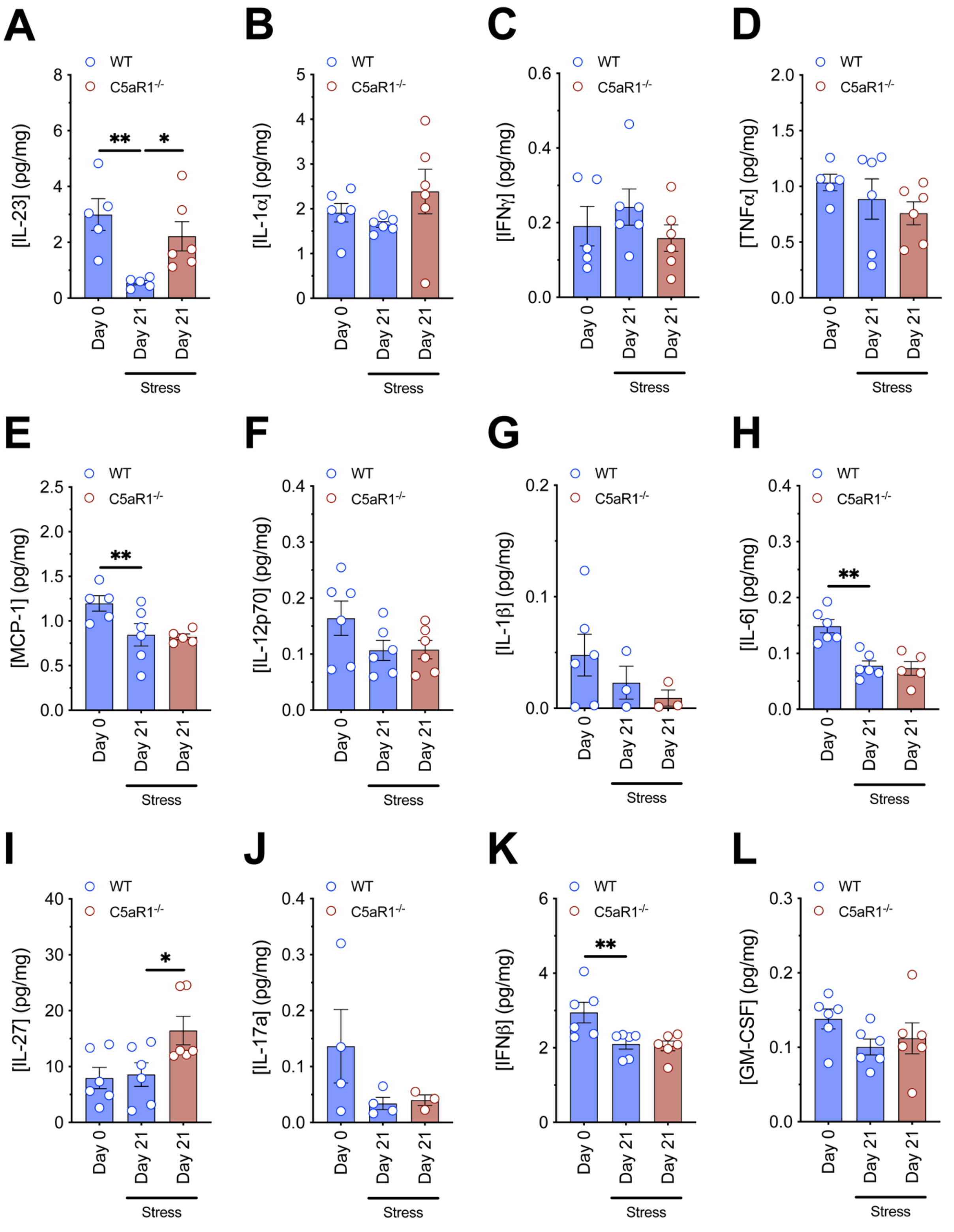

Supplementary Figure 3. Hippocampal interleukin-23 (IL23; **A**), IL-1α (**B**), interferon gamma (IFNγ; **C**), tumor necrosis factor alpha (TNFα; **D**), monocyte chemoattractant protein 1 (MCP-1; **E**), IL-12p70 (**F**), IL-1β (**G**), IL-6 (**H**), IL-27 (**I**), IL-17a (**J**), IFNβ (**K**), and

granulocyte-macrophage colony-stimulating factor (GM-CSF; **L**) protein expression from wild-type (WT) and C5aR1$^{-/-}$ mice following 21 days of 2 h restraint stress compared to non-stressed control (n = 6 per group). Data are expressed as mean ± SEM, and *p < 0.05, and **p < 0.01.

Supplementary References

1. Spiers JG, Chen HC, Steyn FJ, Lavidis NA, Woodruff TM, Lee JD (2017): Noninvasive assessment of altered activity following restraint in mice using an automated physiological monitoring system. *Stress*. 20:59-67.

2. Wirths O (2017): Extraction of Soluble and Insoluble Protein Fractions from Mouse Brains and Spinal Cords. *Bio Protoc*. 7:e2422.
